# The Use and Abuse of Amalgam

**Published:** 1883-07

**Authors:** A. H. Thompson

**Affiliations:** Topeka, Kansas.


ARTICLE II.

### THE USE AND ABUSE OF AMALGAM.

BY A. H. THOMPSON, D. D. S., TOPEKA, KANSAS.

(Read before Kansas State Association).

Whatever may be said against amalgam as a filling material on the score of ethics, however much we may deprecate its extensive and growing employment, and regret the consequent decadence of high art in the execution of gold operations throughout the profession, the gigantic and stubborn fact remains that its employment for filling teeth is most extensively prevalent, and that it is on the increase. Our most strenuous opposition cannot take from the quantity already prevailing, nor prevent its growth. The presence of amalgam with us is a tremendous fact, which we must accept—and accepting, must study. It is a great factor in the dental economy of the day, which cannot be ignored; and we are unable to prohibit its use if we would, even if it were as pernicious as some would have us believe.

On purely ethical and artistic grounds, objection is made to amalgam, on the score of its being an ignoble, filthy, degrading material, unfit for the touch of the members of a high and noble calling. The protests of the all-gold critics are aimed not so much at its practical value and tooth-conserving qualities, as against its plebeian origin and nature. The cry of the golden æsthetics against it arises in fact from a caste prejudice, an ostracism of this pariah amongst filling materials on account of its low pedigree. We are told that no dentist worthy of the name will use such a degrading material, that it compromises not only his professional, but his personal dignity and purity, that the miserable ease with which it is studed into cavities tends only and forever downwards, with the result of rapid depreciation of operative skill, the decadence of art, and the retarding of the spread of the artistic, propaganda, which is the cause dear to the masters in operative art.

But we are not told that we should abandon it because it is worthless practically, that it cannot save teeth, that its conserving qulity is a failure. Its practical value to the dental profession stands unchallenged ; and therein is its strength—the one cause of its universal use to-day, and its increasing popularity. In point of fact, it saves more teeth than any other filling material—taking teeth of all grades as they come to the dentist, and taking all grades of operators as they occur in all ranks of the profession. The poor workman and mediocre operators—whose name is legion, and who coustitute the vast majority of the dental rank and file to-day—can make better amalgam filling than gold filling, in all qualities of teeth as they meet them in daily practice ; and with it they can save more teeth for their patients than they could possibly do with gold. For this reason amalgam saves more teeth in this country to-day than gold and is more generally useful.

Out of the great army of practitioners in the United States of all grades and assorted abilities, there is but a small per cent. who are capable of using gold as it should be used, and of saving teeth with it as they should be saved; and a yet larger proportion besides work gold indifferently well —with some success, but not as the highly favored and artistic few. But the great residue use little gold and much amalgam, because they can thereby do better work and more faithful service to their fellow-man—and therein they are right. They can fill teeth with it easily, rapidly, and cheaply—and therein they are right; for it brings serviceable tooth-saving within the reach of the great mases who cannot afford the services even of the ordinary gold operators. Yet in view of this great economical fact, the gold tyrants would utterly prohibit the use of amalgam and compel all these people to go without the services even of the indifferent tooth-saver. The artistic difficulty of working gold is with them a chief recommendation in that it exclude all men of mean degree and low abilities.

It is an indisputable fact of course, that the use of amalgam will not contribute to the development of artistic ability in the working of gold throughout the mass of the profession but there remains also the fact that the mass of the people cannot be brought up to artistic gold prices, and without just compensation high art cannot be developed in any department. It must have its wealthy patrons or it cannot flourish, and especially is this true of artistic tooth-filling. Good gold-filling cannot be performed without gold fees, and the classes of people in this great land, where wealth is so unequally distributed, who can compensate the skillful operator for his outlay, are not sufficient, so to speak, to "go around." There must therefore be a cheaper material that is serviceable and reliable, and amalgam is that material. A general high-art artistic ability in gold work must therefore go down before the cheaper material. These are disagreeable facts, but we must take them as we find them.

And yet amalgam is not necessarily degrading, and the poor operator is not alone in his use of it. A candid and discriminating judgment impels its employment at times, even by the best operators—*i. e.*, by those who are good men, and who have the greatest good of their patients at heart. Thus, conscientious men often find themselves obliged to use amalgam, when they would prefer to use gold. But it becomes a choice between amalgam and extraction. Amalgam performs services that are beyond gold, and saves teeth gold cannot save; and the honest operators will save teeth with amalgam rather than not save them at all. Therein is the judicious and honest practice —the exercise of true eclecticism in the choice of filling materials. Extreme theories of practice are proverbially unsafe and dishonest. We must follow the safe middle path of conscientious, unbiased eclecticism.

In practice it is the peculiar condition or quality of the cavities, or of the teeth, or of their surroundings, that indicate the superiority of amalgam over gold as a conserving medium in given cases. For instance, in very soft, brittle and chalky teeth, accompained by a permanent condition of acidity or ropiness of the saliva, superabundant mucus, lymphatic temperament, etc.—associated, as this too often is, by a chronic habit of neglect of methodic cleansing—it is madness and dishonesty to fill cavities in such teeth, especially when large and difficulty, or when upon the approximal and buccal surfaces of the posterior teeth with gold. Every candid operator will admit this proposition. It is still better to fill every posterior, concealed cavity with amalgam, for it is particularly indicated in these teeth as the most serviceable conserving material. And why? Because the congenital softness, brittleness, and sponginess of the walls and margins of the cavities in teeth of the class described will admit of a better joint, more nearly water-tight, being made with amalgam than with gold. The softness and elasticily of the walls will not possibly permit of perfect condensation of the gold against them, and leakage is inevitable; but the plastic condition of amalgam, taken with the advantage of slight expansion hardening, for all amalgams do expand slightly, a more perfect co-aptation is obtained and a more nearly water-tight joint is made. In addition to these advantages, as if to support and supplant the inherent lack of strength of the tooth and the difficulty of saving it, the ultimate oxidation will assist in its preservation, be it ever so soft, if work is at all well done by rendering the dentos more dense and less soluble. This oxidation is the result of the attack of the acids upon the metals of the amalgam, where they expend their energy instead of attacking the weak dentos, thus creating the oxides and sulphides which protect and harden the tooth. In this, amalgam performs a vicarious and noble duty, and the unsightly blacking proves a blessing. But with gold, the acids attracted by the metal, as all metals in the mouth do attract and condense acids, are concentrated upon the gold and attack the margins of the cavity, while the gold remains exempt from injury, thus performing an injurious office. In very soft teeth, we hold it is better, therefore, to fill all unexposed cavities with amalgam, but in those of a little harder grade, medium soft structures, which are well cared for (which is an important factor and to be considered in all dental operations), cavities in positions which are kept well cleaned, or on grinding and buccal surfaces, should be filled with gold. Such cavities are the first in lowest grades of teeth, the first on the scale as we approach the better organized, which will admit of gold. All cavities possible should, of course, be filled with gold, and the possibilities begin with the cavities we have described but the approximate cavities in these teeth, especially the large ones, should be filled with amalgam, which is a proper discrimination. All exposed approximal cavities might be filled with tin or amalgam and faced with gold on the masticating margins. It is an abuse of amalgam which has justly brought much of the condemnation of the gold-workers upon it, that it is employed too often where gold should and could be used, because gold is *per se* a better and more elegant and durable material, as a material. Gold is the first and best filling material, but it is incapable of saving all teeth. Amalgam then come in to support it and perform the services it cannot, and saves the teeth it cannot save. It is an aid to gold—an assistant—which attends the possibilities of tooth-saving.

In the interior teeth, amalgam is inadmissible, except, where the teeth are of very dark structure and belong to very filthy persons. We have all meet such cases ; and certainly neither teeth nor their possessors deserve gold. Sometimes, however, a non-oxidizable amalgam can be used in better for covering a grooved, cutting edge, with weak walls to advantage ; or in cavities beneath gums or on the posterior sides of the incisors. As a rule, however, it cannot be employed in visible positions. As a country woman recently remarked, "She did not want any of that blue filling in her front teeth," and we agreed with her. In teeth of medium structure, of course gold must be used in the interior teeth, and also in the medium soft if well cared for, as the conical shape of these teeth facilitates cleaning and preservation. In those which are very soft and when very frail, however, some other of the plastics must be used. Amalgam is also indicated in teeth of the better qualities, where the walls are thin and weak from excessive decay. The danger of breaking under the gold while filling, or the almost certainty of breaking away from the gold afterwards point to the rejection of gold and the substitution of amalgam as the wisest thing to do.

Amalgam will support frail walls ; will not spread under mastication (as gold in bulk will do and strain the walls) and the oxidation, though unsightly, will aid in the preservation of the decaying dentine. It will avoid the suffering incident to protracted gold operations, which is a matter not to be disregarded, when we think of the long train of evils likely to follow upon a severe nervous prostration. Besides, the large amalgam fillings are almost certain to endure longer than their duplicates in gold, even when performed by the very best of operators. To prove this, we need only refer to the fact noticed by all of us in practice, that very large amalgam fillings, which have endured and been in active service for from ten to twenty years, are of not uncommon occurrence, but that large gold fillings which have been durable and serviceable in similar positions, are conspicuous by their rarity. This is a fact which needs no comment; it is at once suggestive and descisive.

Cavities in inaccessible and inconvenient positions, which cannot by any possible exercise of engenuity be filled with gold amalgam filling is better than poor gold filling in any location. It is possible in difficult places to make a better operation, to reduce the amount of suffering required and render better service, than by attempting to use gold and make but an indifferent operation of it at best. We need but refer to those cavities high up on the neck of molars approximate especially—or to those difficult and most unsatisfactory fillings upon the buccal faces of the wisdom teeth. Indeed, wisdom teeth, taking them as they come, are more likely to be better preserved with amalgam than with gold, because of their inherent softness, the impossibility of operating upon them satisfactorily, the pain inflicted, and endurance required.

Teeth which are so seriously affected with calculus, Rigg's disease, protracted ulceration or any other lesion which renders them loose and insecure, should not have their usefulness or, indeed, their very existence endangered by the force and appliances necessary for filling with gold. Amalgam is easier, safer, and fully as serviceable in such teeth. The same argument may apply to large fillings over pulps which have previously been exposed. They must be handled with care, for a pulp once inflamed is never in normal health afterwards; and the force of gold filling in the cavity or in the tooth-socket may arouse a stubborn pulpitis or peri-odontitis. Deciduous teeth are also better preserved with amalgam than with gold, on account of the unusual and normal softness. It is easier of introduction and saves the little patients hours of needless suffering. The permanent teeth during childhood and adolescence are also better preserved by being filled with amalgam, on account of their softness during growing years. Gold nearly always fails in childhood, but on reaching adult age, can be inserted with safety and more assurance of success. Then the amalgam can be removed and replaced with gold, thus allowing it to perform a temporary service, and a better one than gold was capable of. During the growing years the oral fluids seem to be acid and corrosive, but on attaining maturity, they usually become neutral or alkaline, and the enamel seems to acquire a density and polish unknown before, and then it is safe to fill with gold,

Patients whose physical condition is such as to render them unfit to safely endure protracted or painful operations should have amalgam used as largely as possible. Very frequently the lack of health and strength, either from a chronic malady or convalescence after accute disease, pregnancy, etc., is such as to forbid the infliction of much pain; and disregard of these indications of insufficient strength will sometimes precipitate a latent disease or catastrophe, or a great nervous disturbance which become chronic, if indeed it does not imperil life itself. For invalids of all kinds we must exercise the most judicious and careful discrimination to the end of not inflicting more evil than we cure. The achievement of fine operations will not always justify the risk to the patient, and besides, on recovering good health, the amalgam can be removed and gold introduced.

We must notice finally, those occasions where to use amalgam is to abuse it. The principal, one of course, is to fill teeth with it which indicate, by their texture and quality that they should be filled with gold and that they would respond fondly to its conservative power. This is the point where the mediocre operator fails of his high calling and abuses his trust He therein abuses amalgam. But yet again, he is justifiable—he employs a material he can manipulate most successfully and with which he can save the most teeth, and therein he is right. But again, the best of us abuse amalgam by using it in teeth worthy of gold, on the score of economy to the patients, who is unabled to compensate us for gold operations. We are obliged to use amalgam oftimes out of charity, for teeth of the poor must be saved and they cannot pay for high-class operations. The benevolent practitioner, with a flourish of large heartedness, has frequently urged that we should fill the teeth of such patients with gold at any rate for charity's sake ; that the gratitude of an appreciative soul and the approval of one's conscience is the best reward, etc. But there are few of us who could stand the rush of charitable business that such generosity would entail, much as we might admire the sentiment and desire to put it into practice. Besides, the men who are most vociferous in advising this benevolence are the last to practice it. The ordinary practitioner usually has as much charity practice intentional and otherwise, as his strength and means will allow. So it is we are forced into a position of apparent compromise, by being obliged to fill teeth with amalgam which should be filled with gold ; but it is not a compromise if we advise the patient that the amalgam should be considered tentative, and that as soon as convenient he should have the amalgam fillings removed and gold inserted, thus reducing it to a temporary expedient. If the time never comes when he can afford this, his teeth will still be preserved for service and benefit.

In the use of amalgams, as in all other things in practice, we must exercise an intelligent, careful, and candid discrimination, praying and striving to free ourselves from prejudice and the greatest good to the patient, regardless alike of *New Departure* proclamations as conservative anathemas.

Although a reckless candor prevails throughout this paper, and although the writer is cognizant of the denunciations which have been hurled at amalgam and amalgam advocates, he cannot but believe he has voiced the sentiments of almost every conscientious operator and depicted his every-day practice ; though the fear of criticism and the dread of the anathemas of the Inner Circle of Conservatives, have deterred him from avowing his beliefs and practice.—*Missouri Dental Journal.*

